# Time-Sampled Population Sequencing Reveals the Interplay of Selection and Genetic Drift in Experimental Evolution of *Potato Virus Y*

**DOI:** 10.1128/JVI.00690-17

**Published:** 2017-07-27

**Authors:** Denis Kutnjak, Santiago F. Elena, Maja Ravnikar

**Affiliations:** a Department of Biotechnology and Systems Biology, National Institute of Biology, Ljubljana, Slovenia; b Jožef Stefan International Postgraduate School, Ljubljana, Slovenia; c Instituto de Biología Molecular y Celular de Plantas (IBMCP), Consejo Superior de Investigaciones Científicas-Universitat Politècnica de València, València, Spain; d Instituto de Biología Integrativa de Sistemas (I^2^SysBio), Consejo Superior de Investigaciones Científicas-Universitat de València, València, Spain; e The Santa Fe Institute, Santa Fe, New Mexico, USA; University of Maryland, College Park

**Keywords:** *Potato virus Y*, experimental evolution, genetic drift, natural selection, sRNA deep sequencing

## Abstract

RNA viruses are one of the fastest-evolving biological entities. Within their hosts, they exist as genetically diverse populations (i.e., viral mutant swarms), which are sculpted by different evolutionary mechanisms, such as mutation, natural selection, and genetic drift, and also the interactions between genetic variants within the mutant swarms. To elucidate the mechanisms that modulate the population diversity of an important plant-pathogenic virus, we performed evolution experiments with *Potato virus Y* (PVY) in potato genotypes that differ in their defense response against the virus. Using deep sequencing of small RNAs, we followed the temporal dynamics of standing and newly generated variations in the evolving viral lineages. A time-sampled approach allowed us to (i) reconstruct theoretical haplotypes in the starting population by using clustering of single nucleotide polymorphisms' trajectories and (ii) use quantitative population genetics approaches to estimate the contribution of selection and genetic drift, and their interplay, to the evolution of the virus. We detected imprints of strong selective sweeps and narrow genetic bottlenecks, followed by the shift in frequency of selected haplotypes. Comparison of patterns of viral evolution in differently susceptible host genotypes indicated possible diversifying evolution of PVY in the less-susceptible host (efficient in the accumulation of salicylic acid).

**IMPORTANCE** High diversity of within-host populations of RNA viruses is an important aspect of their biology, since they represent a reservoir of genetic variants, which can enable quick adaptation of viruses to a changing environment. This study focuses on an important plant virus, *Potato virus Y*, and describes, at high resolution, temporal changes in the structure of viral populations within different potato genotypes. A novel and easy-to-implement computational approach was established to cluster single nucleotide polymorphisms into viral haplotypes from very short sequencing reads. During the experiment, a shift in the frequency of selected viral haplotypes was observed after a narrow genetic bottleneck, indicating an important role of the genetic drift in the evolution of the virus. On the other hand, a possible case of diversifying selection of the virus was observed in less susceptible host genotypes.

## INTRODUCTION

Mutation, recombination, natural selection, and genetic drift are the processes that shape the diversity and evolution of virus populations. Due to their error-prone replication, RNA viruses mutate rapidly and thus exist within hosts as mutant swarms (also referred to as quasispecies), derived from ancestral infecting genomes (1, 2). Fast replication, coupled with high mutation and recombination rates (3–5) and large populations, enables RNA viruses to quickly adapt to changing environments. These properties also render them interesting for studies of basic evolutionary processes, e.g., mutation, selection, and genetic drift. Whereas selection describes the competition between entities with different fitness generated by mutation within the population, random genetic drift represents stochastic changes in population structure due to limited population sizes. The latter are, for viruses, often a consequence of bottlenecks during transmission and within-host movement (6). Additionally, it has been suggested that the evolution of large diverse populations, like viral ones, is strongly affected by selection of linked mutations on the haplotype and the interactions between different haplotypes (i.e., clonal interference) in the population (7).

For viruses, host switches can represent important ecological changes, posing different, and sometimes conflicting, selection pressures on the virus population (8). These selective forces can leave an imprint in the viral genomes, which can show parallel fixed mutations in viral lineages independently evolved into the same host species (9, 10). Moreover, evolutionary experiments have shown different degrees of parallel evolution after the serial transmission of viruses in different environments, such as adaptation of *Chikungunya virus* (CHIKV) to different vector species (11), adaptation of *Vesicular stomatitis virus* (VSV) to different mammal cell types (12, 13), adaptation of *Influenza A virus* (IAV) to the presence of a drug (14), and others (15, 16). Recently, evidence for host specific convergent mutations were shown also during the experimental evolution of *Tobacco etch virus* (TEV) in different ecotypes of the same species (17).

Detection of mutations and characterization of changes in viral mutant swarms during virus evolution experiments have dramatically improved with the advent of next-generation sequencing (NGS), enabling to detect mutations present at even very low frequencies within the population (18). Most of the virus evolution studies rely on snapshots of viral diversity at a particular time point. Such an approach has limitations to detect selection patterns (relying on the ratio between observed synonymous and nonsynonymous mutations). Implementation of time-sampled approaches in evolution experiments (14, 18–21), on the other hand, enables to follow trajectories of mutations in a larger time frame and thus to infer some population genetics parameters, such as selection coefficients (*s*; a measure of the strength of natural selection operating on a given viral genotype) and effective population size (*N_e_*; a measure of the strength of genetic drift) (14, 19).

In this research, we have focused on 1 of the 10 most important plant viruses, *Potato virus Y* (PVY; genus *Potyvirus*, family *Potyviridae*) (22), which causes a substantial yield loss in potato production worldwide (23). PVY is a single-stranded positive-sense RNA virus, comprising a high diversity of different recombinant strains. Specifically, we have focused on the recombinant strain NTN, which causes the potato tuber necrotic ringspot disease (PTNRD). The response of potato to infection with PVY has been extensively studied at different levels (24–27), and it was shown that hormone signaling has an important role in perception and response to the virus infection, salicylic acid (SA) representing one of the hallmarks of the response (28–30). Disturbance in SA signaling was shown to affect accumulation of the virus and the appearance/severity of the disease symptoms (26, 28). In the present study, we aimed to (i) elucidate whether differentially susceptible potato genotypes have an effect on PVY population structure and (ii) get new insights into the mechanisms shaping the evolution of the virus within different genotypes of the same host species. To reach these goals, we have performed experimental evolution of PVY in potato plants accumulating SA (representing a less permissive environment) and potato plants depleted in SA (thus representing a more permissive environment).

**FIG 1:**
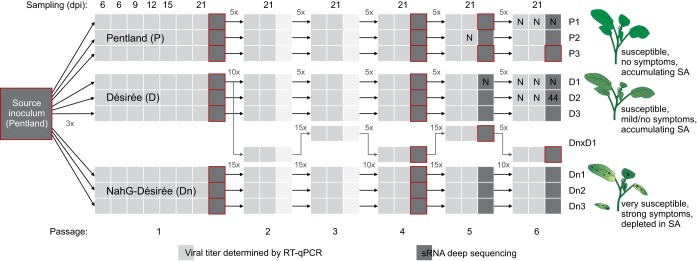
Setup of the evolution experiment. Three lineages of three different potato genotypes (Pentland squire, Désirée, NahG-Désirée) were inoculated with the same PVY inoculum (derived from the pool of systemically infected cv. Pentland squire plants [big square]). Six serial passages of the virus were performed for each lineage. For one of the lineages, the host plant genotype was alternated between Désirée and NahG-Désirée (lineage DnxD). Small squares represent individual plants, which were sampled either for viral titer determination by RT-qPCR (light gray) or for population sequencing by Illumina sRNA deep sequencing (dark gray; the same plant was also used to infect the consecutive generation of plants). Above the scheme, sampling time points are designated in days postinoculation (dpi). Coefficients next to the arrows designate the dilution coefficient used to approximately normalize the virus titer in inocula in each passage. Samples negative for virus are labeled “N.” In the 6th passage, one of the samples was sampled later (44 dpi) than others and is indicated on the scheme by the number 44. Red outlines identify the samples included into the selection analysis with WF-ABC (some of the samples were excluded due to the observation of very narrow genetic bottlenecks).

We have recently demonstrated that virus-derived small interfering RNAs (vsiRNAs) cover the complete genome of PVY and accurately describe the population structure of PVY in potato (31), showing it to be highly similar to the one observed by sequencing RNA from purified virions. By combining the high resolution of Illumina small RNA (sRNA) deep sequencing and a time-sampled experimental evolution approach, we have now tracked the dynamics of PVY populations during serial passages of 10 separate lineages in 3 different potato (Solanum tuberosum L.) genotypes (Fig. 1): Pentland squire (P), Désirée (D), and NahG-Désirée (Dn). P is a tolerant cultivar, accumulating virus but not showing any symptoms; D is susceptible for PVY^NTN^, sporadically shows symptoms after infection, but is asymptomatic in our experimental setup; Dn is a transgenic genotype derived from D transformed with the *nahG* gene (salicylate hydroxylase), which converts SA into catechol; it is susceptible to PVY and expresses strong symptoms in the form of necrotic lesions on the leaves (28). The source inoculum used to initiate all of the evolving lineages was derived from P plants (Fig. 1). Rather than starting from a cloned viral genome, we have constituted a virus inoculum with high genetic variability, which allowed us to study the relative contribution to virus adaption and diversification of standing genetic variation in the starting population and of new variation created during the evolution experiment. We have observed signatures of selection and genetic drift and their interplay in the evolution of PVY. Additionally, we have detected diversifying evolution of the virus in the less susceptible host, i.e., in the genotypes accumulating SA.

## RESULTS

### An unexpected drop in viral titer in one of the serial passages resulted in a narrow genetic bottleneck.

**FIG 2:**
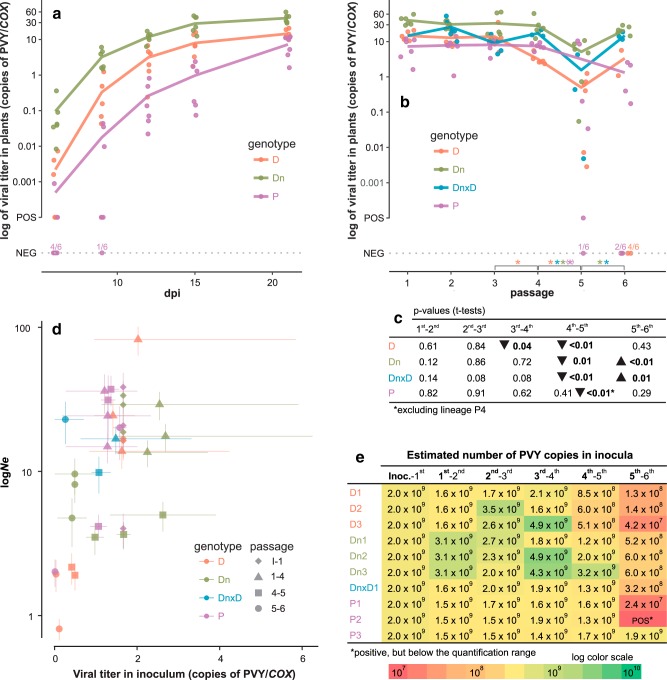
Viral titers and effective population sizes. (a and b) Viral titers measured at 6, 9, 12, 15, and 21 dpi for the 1st passage (a) and at 21 dpi in each of the 6 passages (b). Samples positive for PVY but below the quantitative range are included in the plots at the level labeled “POS,” and negative samples are included at the level labeled “NEG” (for each genotype, the ratio of the number of negative samples to the total tested is shown above the “NEG” dotted line). Lines connect average values, which are calculated only for positive samples. Asterisks above the *x* axis (b) designate statistically significant differences (*P* = 0.05) of viral titer means of consecutive passages for each genotype (color coded). For genotype P, the difference was statistically significant after removing one of the lineages (P3, for which the titer was not reduced) from the analysis (indicated by an asterisk in parentheses). (c) *P* values of the corresponding *t* tests are presented below the line graph, and triangles next to the numbers mark significant increases or decreases in viral titer. (d) Viral titers in the inocula plotted against log-transformed estimated effective population sizes (*N_e_*). Points represent average values, and horizontal lines connect minimum and maximum measured values for the viral titer for a specific lineage/passage, whereas vertical lines represent standard deviations of estimated *N_e_*. (e) Estimated numbers of PVY copies in 250 μl of inocula (the amount used to infect one plant) for different passages are shown and visualized as a heat map with a logarithmic color scale. Different individuals were used for the estimation of viral titer on one hand and population sequencing on the other (Fig. 1). In all panels, the host genotypes are color coded. In panel d, different symbols indicate different passages.

We have first measured virus accumulation in the three different potato genotypes by measuring its titer 6, 9, 12, 15, and 21 days postinoculation (dpi) in the first passage. Virus accumulation was correlated to the severity of the disease symptoms on the different genotypes; the genotype with the most-severe disease symptoms (Dn) accumulated the largest amount of the virus and the tolerant cultivar (P) the smallest amount. Virus titers approached a plateau in all three genotypes 21 dpi, the time point at which sampling for virus population sequencing was performed. The differences in virus accumulation in the first passage (21 dpi) between the three cultivars were significant (pairwise *t* tests, *P* < 0.05) and ranged between 1 and 2 orders of magnitude, the difference between the smallest values (P) and biggest values (Dn) being around 60-fold (Fig. 2a).

The viral titer was then measured after each passage (in all cases at 21 dpi) (Fig. 2b), and it remained relatively stable or only slightly decreased (in D) until the 5th passage, where we observed a significant drop in virus titer in all of the potato genotypes. Pairwise *t* tests comparing consecutive pairs of passages showed that viral titers were significantly reduced (*P* < 0.05) from the 4th to the 5th passages in 9 of 10 lineages (Fig. 2b). Consequently, the virus titers were also reduced in the inocula used to infect the subsequent passage of plants in the experiment (Fig. 2d). The underlying reason for the observed drop is unclear. We hypothesize that this could be an experimental artifact, likely originating from the plant inoculation process or from an unexpected variation in some uncontrolled environmental factor affecting only this particular passage. The virus titer for the lineage DnxD1 (alternating host plants between D and Dn) fluctuated according to the host cultivar (Fig. 2b, blue line).

Small RNA deep sequencing results were obtained for a total of 43 samples from different experimental passages. For all of the samples, vsiRNA reads covered the complete genome of PVY at high or very high depth (see Materials and Methods; see also Data Set S1 in the supplemental material for details), which enabled reliable detection of single nucleotide polymorphisms (SNPs) in all of the samples. All of the viral lineages in the experiment were derived from the same starting inoculum (derived from infected P plants), which was also sequenced and used as a starting point for the analyses. The viral population in the starting inoculum was highly diverse, containing in total 85 variable sites (64 synonymous and 21 nonsynonymous; see Data Set S3 in the supplemental material for details).

SNP data were then used to calculate nucleotide diversities (π) of the virus populations and to estimate the effective population sizes (*N_e_*) as well as SNPs' selection coefficients (*s*) from temporal information using a Wright Fisher approximate Bayesian computations (WF-ABC) approach (19). *π* is a measure of the heterogeneity of virus populations; *s* represents a measure of relative fitness of the competing individual against the alternative individuals and as such reflects the strength of natural selection; *N_e_* is an estimator of the strength of genetic drift, since it represents the number of virus genomes contributing directly to the progeny, being determined by the size of the population bottlenecks (6).

To determine if the observed drop in virus titer in the 5th passage was reflected also in a narrower genetic bottleneck, the first part of the WF-ABC analysis (*N_e_* estimation) was performed separately for pairs of time points (inoculum and 1st passage; 1st passage and 4th passage; 4th passage and 5th passage; 5th passage and 6th passage). The estimations of *N_e_* for pairs of time points showed a pattern similar to the one observed in viral titer measurements, suggesting that the strong drop in viral titer at the 5th passage was reflected in lower *N_e_* values estimated for the corresponding viral populations in this time point (Fig. 2c).

### Nucleotide diversity (*π*) values corroborate the observed genetic bottleneck and indicate the involvement of selection in shaping viral populations.

**FIG 3:**
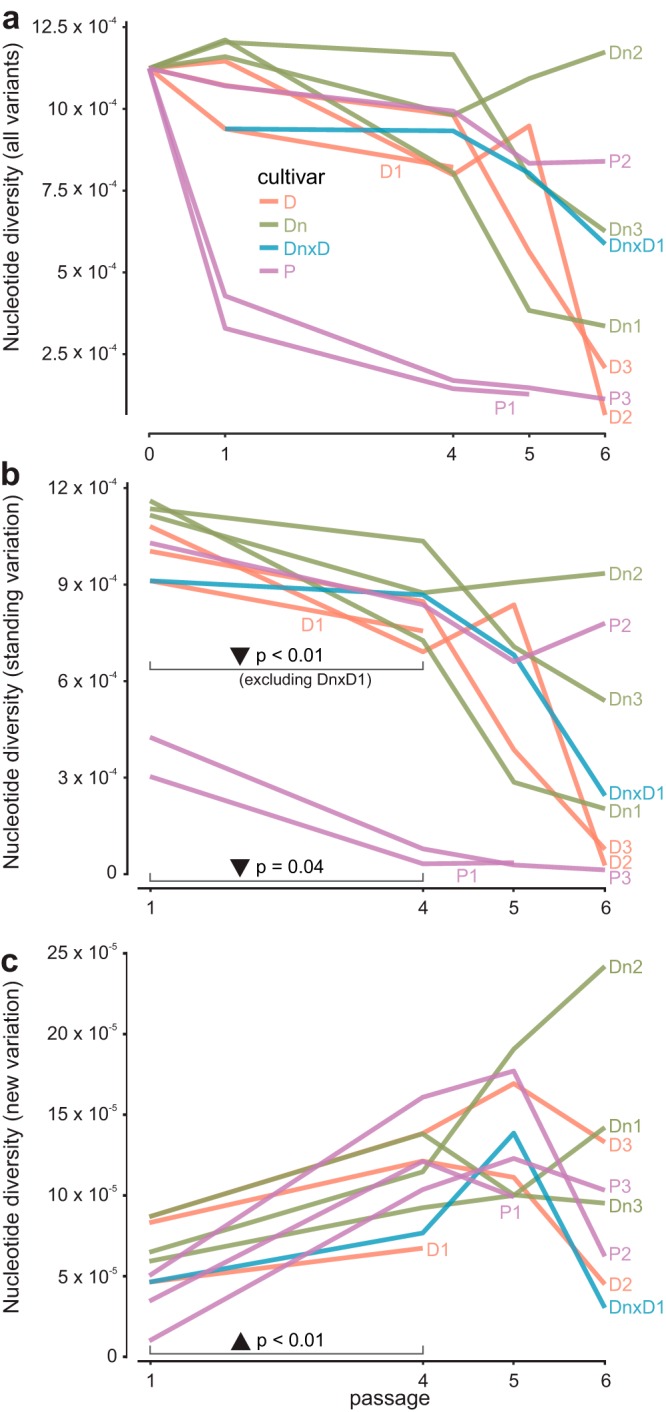
Virus population diversity dynamics. The average population nucleotide diversity (*π*) was calculated for all SNPs (a), SNPs present already in the source inoculum (standing variation) (b), and new spontaneous mutations (new variation) (c). Lines are connecting calculated π values through time points for each lineage; host genotypes are color coded in all three panels. (b, c) Results of the pairwise *t* tests for the 1st and the 4th passages are shown, indicating a significant decrease in *π* values for standing variation (b) but a significant increase in *π* values for new variation (c).

We estimated the changes in the diversities of the virus populations by calculating populations' nucleotide diversities (*π*) from SNP data for each sequenced sample. The estimated *π* values also reflected a genetic bottleneck in the 5th passage of the experiment. For most lineages, nucleotide diversities remained relatively stable or decreased slightly until the 5th passage, where we observed a disturbed pattern (Fig. 3a), likely resulting from strong genetic drift, a consequence of severely reduced *N_e_*. As a contrast to the predominant pattern, a strong decrease in nucleotide diversities was detected for lineages P1 and P3 immediately after the first passage (Fig. 3). Detailed inspection of SNP trajectories in these two lineages showed that a large group of SNPs was quickly fixed and others were eliminated from the populations, indicating the presence of a strong selective sweep.

To get further insights into the dynamics of the viral populations, total variability was subsequently separated into two components: standing variation (SNPs already present in the source inoculum) and new variation (SNPs appearing during the evolution experiment). Separate nucleotide diversity analyses of standing and new variations showed that standing variation was decreasing until the 4th passage of the experiment (Fig. 3b) in most lineages, whereas nucleotide diversity (*π*) for new SNPs was increasing (Fig. 3c). Pairwise *t* tests showed significant differences between the means of *π* values between the 1st and the 4th passages for both cases (see Fig. 3b and c). Only in lineage DnxD1, in which the host was alternated between D and Dn, did standing variation remain very stable from the 1st to the 4th passages, possibly indicating conflicting selection pressures in the two genotypes, which prevented fixation of specific SNPs. Again, the strong influence of genetic drift disturbed the predominant pattern in the 5th passage, where we noted a drop in *π* values in most of the lineages also for new variation (Fig. 3c). However, interestingly, in lineage Dn2 nucleotide diversities for new SNPs sharply increased in this passage and kept rising also in the 6th passage (Fig. 3c). A more detailed inspection of the pattern of variation for this lineage shows that several new nonsynonymous mutations start to rise in frequency in this passage, likely indicating the strong impact of selection after the observed bottleneck.

### Selection analysis and clustering reveal groups of linked SNPs and enable the reconstruction of haplotypes in the source inoculum.

**FIG 4:**
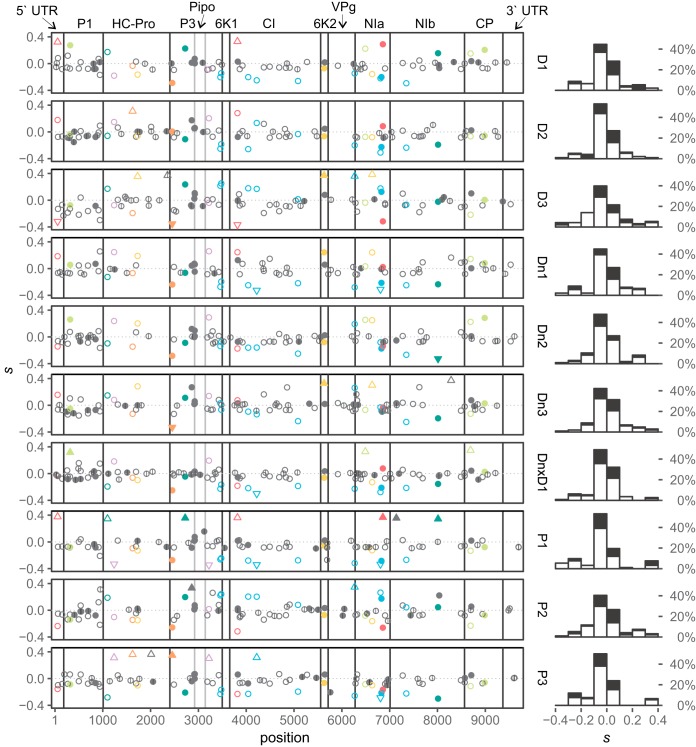
Distribution of SNPs on the PVY genome and their selection coefficients (*s*) in different lineages. *s* values were estimated using the WF-ABC approach, excluding the time points with observed severe bottlenecks (Fig. 1). On the left, posterior means of estimated selection coefficients for SNPs in different lineages are plotted across the PVY genome. Empty signs, synonymous SNPs; filled signs, nonsynonymous SNPs; triangles, SNPs for which neutrality was rejected (95% HPD interval does not include 0); ticks within the signs mark the new spontaneous mutations (not present in the source inoculum). Colors correspond to 7 groups of SNPs identified by clustering analysis (Fig. 5); SNPs that were not assigned to any group are in gray. On the right side, distributions of posterior means of estimated *s* of SNPs for each lineage are shown; white surfaces represent synonymous mutations and black surfaces nonsynonymous mutations.

To better understand the impact of selection operating on viral populations in different lineages, we conducted the second part of the WF-ABC analysis: estimation of selection coefficients, *s*, for individual SNPs; SNPs could be neutral (*s* = 0; selection will not affect SNPs frequency), beneficial (*s* > 0; selection will cause an increase in the SNP frequency), or deleterious (*s* < 0; selection will cause a decrease in the SNP frequency). For the majority of SNPs, neutrality was not rejected. For 1 to 11 SNPs per lineage, neutrality was rejected using our stringent statistical criteria (described in Materials and Methods). We have observed loci under both strong positive and negative selection. The latter could be observed since the source inoculum in this experiment contained highly diverse PVY populations, enabling selection to fix some SNPs while removing others. No new SNPs under significant negative selection have been detected, likely due to their quick elimination from the population right after they were generated by mutations. One new SNP with a significantly positive *s* value was detected. The results showed that the distributions of SNP selection coefficients had similar shapes for all 10 lineages (similar fractions of SNPs were assigned as neutral, beneficial, or deleterious in each lineage) (Fig. 4, right panel). Plotting *s* values of individual SNPs onto the PVY genome revealed no apparent clustering pattern in different parts of the genome (Fig. 4, left panel).

The sign of selection (neutral/positive/negative) for a particular SNP differed among lineages, sometimes showing distinct patterns of interaction (positive or negative) between the SNPs. To better understand the patterns of interaction and possible linkage between the observed SNPs, we employed a clustering approach and tried to assign SNPs into groups. Using hierarchical clustering of SNP trajectories (SNPs are clustered together if their frequencies change in a parallel manner over time), several groups of SNPs showing parallelism in their frequency trajectories within the lineages were detected (see Fig. S1 in the supplemental material). The groups of SNPs could theoretically represent SNPs linked onto the same genome (haplotypes) or/and other type of linkage/interactions between the SNPs within the virus population.

**FIG 5:**
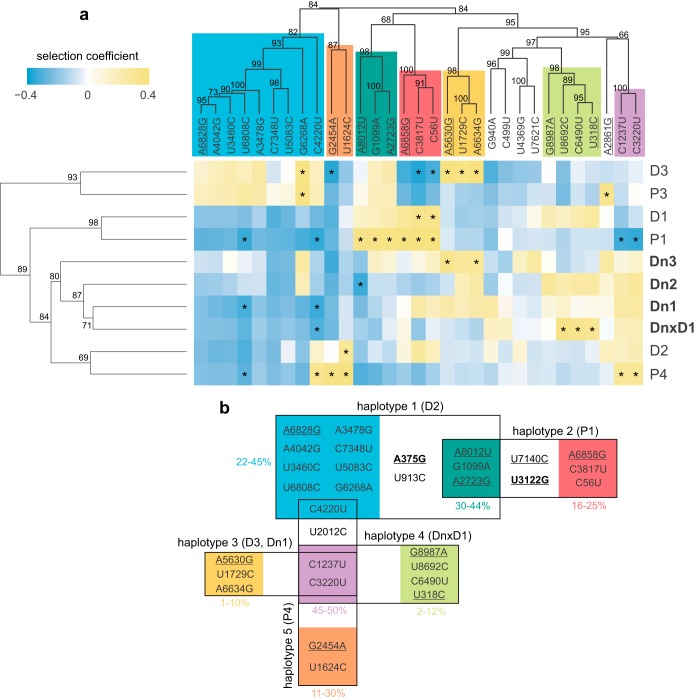
Clustering analysis and haplotype reconstruction. (a) SNPs were clustered into groups based on their inferred selection coefficients in different lineages (columns). Seven recognized groups are color coded. Lineages were clustered based on the selection coefficient of the SNPs (lines); the ones evolved in potato genotypes depleted in salicylic acid are bolded. Only the SNPs occurring in all lineages were included in this analysis. Selection coefficients of SNPs in every lineage are shown in a blue-yellow heat map. Asterisks designate SNPs for which neutrality was rejected (in the respective lineages). The numbers next to the dendrograms' branches represent statistical support of the groups, determined by bootstrapping. Several haplotypes were reconstructed by clustering SNPs' frequency trajectories and observing fixed haplotypes. (b) Reconstructed haplotypes in the source inoculum (lineages in which they were observed are given in parentheses; see Fig. S1 in the supplemental material); boxes are delimiting haplotypes (groups of mutations fixed in the same time point in the respective lineages), and colors are keyed to those of the groups from the clustering analysis shown in panel a. New spontaneous mutations (not present in the source inoculum) are bolded, and nonsynonymous mutations are underlined. Frequencies shown next to the groups are frequencies observed in the source inoculum.

In some of the lineages (lineages P1, P3, D2, and D3; Fig. S1a, c, e, and f in the supplemental material), groups of mutations were fixed (several SNPs reached a frequency of ∼100% in the same population) during the experiment. This necessarily means that those SNPs are all present on the same viral genome backbone, thus directly confirming their linkage to the same haplotype. Therefore, a total of five haplotypes in different lineages (Fig. S1) have been inferred, and finally, their relationship in the source inoculum was reconstructed (Fig. 5b).

Further, the results of the selection study were also analyzed by a second clustering method. Here, we have used estimated selection coefficients of SNPs to cluster (i) SNPs with similar *s* values within the lineage (revealing groups of SNPs under similar selection regime) and (ii) lineages where particular SNPs have more-similar *s* values (revealing groups of lineages under similar selection pressures).

We observed several groups of SNPs (Fig. 5a, column clustering). Seven of these groups represent partial haplotypes, since pairs of these groups formed the haplotypes reconstructed in the first clustering analysis. Integrating the results of the two clustering analyses, we have been able to construct a diagram that represents the grouping of observed SNPs into theoretical haplotypes and their interconnectedness (Fig. 5b). This result suggested the cooccurrence of two major mutant swarms within the source inoculum, the first one containing haplotypes 1 and 2 and the second containing haplotypes 3, 4, and 5. Two haplotypes from the first mutant swarm likely originated from the ancestral haplotype containing the group of mutations indicated by dark green on Fig. 5b, whereas the three haplotypes from the second mutant swarm likely originated from the ancestral haplotype containing mutations indicated in purple in Fig. 5b. A theoretical possibility is that the reconstructed haplotypes were not yet present in the inoculum but emerged later by recombination. However, since we observed at least some of them in several independent lineages, this does not seem a plausible scenario. Based on the observed frequencies of SNPs in the inoculum, one of the mutations, C4220U, likely occurred independently in each of the two mutant swarms or was introduced into one of them by recombination.

### Imprints of diversifying selection in less susceptible genotypes.

We tried to identify specific and similar patterns of selection in different lineages using hierarchical clustering of lineages (based on selection coefficients of SNPs present in all of the lineages, i.e., part of the standing variation). This analysis revealed a structure within the 10 evolved lineages (Fig. 5a, lines clustering). The three lineages evolved in the potato genotype depleted in SA (Dn) were clustered into the same group, including also a lineage in which the host genotype was alternated between Dn and D plants (DnxD1). The statistical support (assessed by bootstrapping) of this group was moderate (80%) and could be biased due to the linkage of some SNPs. Six lineages from the other two cultivars (P and D) did not show any genotype-specific grouping. Principal-component analysis (PCA) of the same data confirmed the grouping observed using a hierarchical clustering approach (results not shown). The observed pattern suggests that the SNPs included in this analysis were assigned more-similar *s* values within the group of Dn lineages than in the other two genotypes, possibly indicating more-homogenous selection pressures within this group or, from the opposite perspective, diversifying selection in the other two genotypes (D and P, both efficient in accumulation of SA).

### Driver mutations and shift in frequency of selected haplotypes after the observed bottleneck event.

Several new SNPs appeared during the experiment, some of them later approaching fixation or being fixed (see Fig. S1 in the supplemental material for a visual representation of SNP frequency dynamics). In P1, the new nonsynonymous mutation U3122G (P3/S240A and PIPO/L68R) appeared in the 2nd passage and could act as a driver mutation, causing fixation of haplotype 2 in this lineage. In lineage P3, synonymous mutation U2012C, which was present in the inoculum at a very low frequency (0.6%), started to rise in frequency and was finally fixed as a part of haplotype 5. The same haplotype started rising in frequency in lineage D2; however, its frequency dropped after the observed bottleneck in the 5th passage and the new nonsynonymous mutation A375G (P1/N64S) emerged on haplotype 1, which was finally fixed in this lineage in the 6th passage. A similar shift between the haplotypes after the bottleneck event was also observed in other lineages. For example, in lineage D3, haplotype 1 was rising in frequency until the 5th passage, after which it was nearly eliminated from the population and haplotype 3 replaced it and approached fixation. Finally, the most striking example of such a pattern is observed in lineage Dn2. Here, haplotype 3 was rising in frequency during the earliest three passages. After the bottleneck, it was removed from the population and haplotype 1 started to rise in frequency. At the same time, 5 new nonsynonymous mutations started to rise in frequency (these SNPs were not included in the selection analysis with WF-ABC, since the time points with observed severe bottlenecks were excluded from that analysis), suggesting the strong impact of selection at these time points. On the other hand, the switch between different haplotypes was not observed in lineage P2: here we observed a reduced frequency of haplotype 1 in the 5th passage, but the same haplotype rose in frequency again in the 6th one.

## DISCUSSION

One of the important limitations of most NGS technologies used is the short length of sequencing reads. This poses a problem when aiming to reconstruct longer sequences, in our case complete viral haplotypes. Several computational approaches have been developed to address this problem (32–36). However, they utilize mostly paired-end reads of substantial length, are hard to optimize, and could produce a high number of false-positive results (37). In our experiment, we used extremely short sRNA reads (21 to 24 nucleotides), which could not be assembled into haplotypes by any of these methods. However, by using a simple clustering analysis of SNP frequency trajectories in time, we have been able to detect several groups of linked SNPs and reconstruct theoretical haplotypes in the inoculum. The approach used was inspired by the analysis of yeast experimental evolution data (38). Refining this approach and maybe connecting it with the above-mentioned ones could lead to better haplotype reconstruction algorithms in future.

Our time-sampled approach allowed us to estimate effective population sizes (*N_e_*) and selection coefficients (*s*) of observed SNPs, using the WF-ABC method (19). The method calculates *N_e_* on the assumption that most of the SNPs in the population are neutral and not linked; thus, since we have observed selection on groups of linked mutations, the method is expected to produce a biased result and would likely underestimate the actual *N_e_* values in our experiment (since selection will cause increase/decrease in frequency of some variants, similar to strong genetic drift, i.e., small *N_e_*), and even more so in lineages P1 and P3, where we observed strong selective sweeps. Nevertheless, the method corroborated the suspected narrow genetic bottlenecks during the 5th passage of the experiment, suggested also by other observations: strong drops in viral titers in the inocula (Fig. 2d) and decreased population nucleotide diversities (both independent of *N_e_* estimates). The association between the virus titer in the inoculum and the estimated effective population sizes seems to be stronger for very low titers (Fig. 2c) and not evident for higher *N_e_* and viral titers, a situation similar to the one observed for another potyvirus, TEV (39). Further, we have used estimated selection coefficients to perform clustering of SNPs (Fig. 5a, column clustering), and the SNP clusters matched well the pattern of haplotypes reconstructed by clustering the SNP frequency trajectories (which was independent of *N_e_* estimates).

We observed strong selective sweeps in two of the lineages (P1 and P3), resulting in rapid reduction of genetic diversity. Since the source inoculum for the experiment was derived from a mix of infected P plants, one could expect the presence of some viral haplotypes already adapted to this genotype in the inoculum (the so-called quasispecies memory [40]). This would allow selection to rapidly fix already-existing well-adapted haplotypes. Such haplotypes would possibly be present in the inoculum at low frequencies, being diluted, when mixing the 24 plants used for inoculum preparation. Indeed, in those two lineages, new (P1) or low-frequency (0.6%, P3) mutations emerged and were finally fixed, maybe acting as driver mutations for selected haplotypes. Even though one of these two mutations was not detected in the source inoculum, it is conceivable that it might have been present there at a frequency below our detection limit, and thus we were unable to separate it from the sequencing errors using our criteria. We used an empirical frequency cutoff criterion, which allowed us to confidently separate real SNPs from sequencing errors. Recent research showed that standing variation in viral populations can contain SNPs at very low frequencies (18). Such very low frequency SNPs could not be told apart from sequencing errors; thus, some of the SNPs, which we classified as new, may indeed be present in the source inoculum at very low frequencies.

Observation of these and other possible driver mutations (in lineages P1, P3, and D2) emphasize the importance of disentangling the linkage between alleles within the viral population. By observing trajectories and/or selection coefficients of separate SNPs, we can count many loci as positively selected in our experiment (e.g., in lineage P1 [Fig. 4]); however, this pattern is likely a consequence of selection operating on linked loci, a process known as genetic draft (7), where one beneficial mutation on a certain haplotype in a population drives this haplotype to fixation.

The narrow genetic bottleneck observed in the 5th passage of our experiment in most of the evolutionary lineages enabled us to further investigate patterns of evolution by observing an interesting interplay between selection and genetic drift. A reduction in genetic diversity was observed in most of the lineages at this time point, and in some cases, haplotypes approaching fixation were eliminated from the population and alternative haplotypes started to rise in frequency after the bottleneck. It has been suggested that genetic drift can translocate virus populations in a hypothetical fitness landscape, from a first local fitness peak to another local peak, by stochastically eliminating part of the viral population, which was approaching the first local peak (6). In such cases, strong genetic drift does not necessarily lead to the maladaptive genotypes, but it helps to move the population from a local fitness peak, where it was stuck, to a different region in the fitness landscape. We observed this phenomenon in our experiment in several lineages as a shift in a positively selected haplotype before and after the bottleneck event (D2, D3, and Dn2). Thus, we hypothesize that the interplay of selection and genetic drift is especially important in very diverse virus populations, where interactions between the individual members of the mutant swarm can affect the fitness of the virus (population) (20). In such cases, strong genetic drift can provide a stochastic subsample of the population, which establishes new interactions and as such translocates the virus in the rugged evolutionary landscape, but also, the reduced complexity of the mutant swarm relaxes the constraints in the fitness landscape, due to the weaker interactions within the population, and enables the rise of new positively selected mutations. The narrow genetic bottleneck observed in the 5th passage of our experiment provided unexpectedly valuable information about the importance of natural selection and genetic drift and their interplay in the evolution of the viral populations. These findings are even more interesting in light of the awareness that viral transmission events in nature almost necessarily involve narrow genetic bottlenecks (6), bringing this experiment setup closer to the expected chain of events in nature.

Finally, we have observed a possible case of diversifying selection in PVY lineages evolved in cultivars efficient in accumulation of SA. Salicylic acid is one of the most important components of the plant response to biotic stress, and it was shown to be involved in the suppression of all the main stages of viral infection (replication and cell-to-cell and long-distance movement) (30). It has been extensively studied in potato-PVY interaction and shown to be a trigger of multiple changes in the activity of downstream metabolic pathways. Recently, research into sRNA expression levels in the same experimental system as ours (PVY + D/Dn) showed that there are considerably more microRNAs differentially expressed in D plants after infection with PVY than in Dn plants (M. Križnik, M. Petek, D. Dobnik, Ž. Ramšak, Š. Baebler, S. Pollmann, J. Kreuze, J. Žel and K. Gruden, unpublished data), indicating a stronger activation of RNA-silencing mechanisms in the presence of SA, a connection suggested also for several other pathosystems (30, 41–43). The pattern of diversification in our experiment can be explained by two non-mutually exclusive scenarios: (i) diversifying selection in the presence of SA and (ii) narrower within-plant virus population bottlenecks occurring in genotypes accumulating SA. The first could have its basis in redundant effects of the SA on the virus, including a strong response and reprogramming of the plant transcriptome (26) and RNA silencing, which could introduce nonhomogeneous selection pressures on the virus population. These could, in combination with the stochastic movement of different viral haplotypes from cell to cell, recently demonstrated for another plant positive-strand RNA virus (44), result in a stronger selection of different haplotypes in different individuals. The second scenario (stronger effect of genetic drift within a plant) could be supported by observed smaller viral titers in P and D plants. Stronger genetic drift could cause more random fixation of haplotypes in these plants than in Dn plants, which are depleted in SA and accumulate higher titers of the virus. In any case, the observed result (higher degree of diversification in the genotypes accumulating SA) suggests a quicker diversification of the virus in somehow less permissive environments. Even though the proposed mechanisms would reduce viral genetic diversity within the host, they could result in an increased diversity of the virus on a higher level (among hosts in the ecosystem), which can hypothetically increase the possibility for the occurrence of new emergent viral isolates.

### Conclusions.

Time-sampled population sequencing data enabled us to reconstruct theoretical haplotypes in a complex starting PVY population mix. We tracked SNPs' and haplotypes' trajectories over time to estimate the impact of selection and genetic drift on the evolution of the virus. Following the general trends of PVY evolution in the experiment, a narrow genetic bottleneck in one of the passages was detected, coinciding with the shift in the selected virus haplotypes in many lineages, indicating the power of genetic drift to change the location of a virus population in the fitness landscape. The fixation of selected haplotypes was often connected with the occurrence and fixation of new mutations, likely acting as drivers for the fixation of groups of SNPs connected to the same haplotype. By comparing the evolution of PVY in different potato genotypes, we detected an imprint of diversifying selection of the virus in less susceptible genotypes, which are efficient in accumulation of SA. Finally, all of these findings are important for understanding the mechanisms that govern the evolution of PVY, as well as other viruses, e.g., how the interplay of selection and genetic drift shapes the genetic structure of the viral populations and how differences in host response to infection affect this interplay.

## MATERIALS AND METHODS

### PVY experimental evolution setup and sampling.

The potato plants used in the experiment were first propagated in stem node culture (*in vitro*). Fourteen days after node segmentation, they were transferred to soil in a growth chamber and kept at 21 ± 2°C in the light and 18 ± 1°C in the dark, at a relative humidity of 75% ± 2%, with 70 to 90 μmol/m^2^/s^2^ radiation (L36W/77 lamp; Osram, Germany) and a 16-h photoperiod. After growing in soil for 28 days, they were either used to prepare the inoculum (systemically infected plantlets) or infected with the inoculum (healthy plantlets) as described below.

Twenty-four Solanum tuberosum L. cv. Pentland squire plants systemically infected with PVY (strain NTN; isolate NIB V 151) were used to prepare the source inoculum. At the same time, two leaf tissue discs (*r* = 5 mm, ∼15 mg per disc) were sampled from each plant, pooled, and frozen for RNA isolation. For inoculum preparation, the rest of the PVY-infected plant material was ground in 0.04 M sodium phosphate buffer with 0.01 M sodium diethyldithiocarbamate (DIECA) (Sigma-Aldrich) in a 1:3 (wt/vol) tissue-to-buffer ratio. This inoculum was used to infect 24 plants per three different potato genotypes (Pentland squire [P]; Désirée [D]; NahG-Désirée [Dn]; the latter being a transgenic cultivar depleted of salicylic acid). The first three fully developed leaves of each plant were rub-inoculated with carborundum (0.062 mm) (VWR International) using ∼75 μl of inoculum per leaf. Inoculum was washed from the inoculated leaves after 10 min, and noninoculated leaves were protected from splashing during the washing step. For the first generation of plants, sampling was performed 6, 9, 12, 15, and 21 dpi by taking one disc of leaf tissue from the first three noninoculated leaves per each plant. Six plants per cultivar per time point were sampled (some of the plants were sampled twice). The plant material was immediately frozen for subsequent RNA isolation. At 21 dpi, an additional three plants per cultivar were harvested and their tissue was equally divided in two. One half was frozen and later used for RNA isolation and next-generation sequencing (NGS). The other half was stored at −20°C and later used to prepare inoculum for infection of the next generation of plants. Each of these nine plants (three per cultivar) was used to initiate a lineage in the evolution experiment, plus an additional lineage in which the host cultivar was alternated between D and Dn genotypes, adding up to 10 lineages in total.

Six serial passages were performed for each lineage (Fig. 1). For each new passage (from the 2nd passage on), eight plants per lineage were inoculated following the procedure described above. Virus titer was determined (see below) for the leaf disc samples from 21 dpi, and this information was used to appropriately dilute the inocula, so that all inocula contained a similar concentration of the virus (Fig. 1). All the inocula were sampled and frozen. From the 2nd passage on, leaf tissue discs were sampled at 6 (2 plants per lineage), 9 (1 plant per lineage), 12 (1 plant per lineage), 15 (1 plant per lineage), and 21 (2 plants per lineage) dpi. At 21 dpi, one plant per lineage was harvested and used as inoculum for the next generation of plants and NGS (with the sampling procedure described above).

### Virus titer determination.

Virus titer was determined for leaf tissue disc samples sampled at 6, 9, 12, 15, and 21 dpi for the 1st passage and at 21 dpi for the 2nd to the 6th passages.

Total RNA was isolated from plant tissue discs (∼45 mg) using the MagMAX-96 Total RNA Isolation kits (Life Technologies, USA) and the KingFisher Purification system (Thermo Scientific) according to the manufacturer's instructions for plant samples. Homogenization of the plant material was performed using the FastPrep-24 instrument (MP Biomedicals, USA).

Absolute quantification of virus titer was performed by reverse transcription-quantitative PCR (RT-qPCR) and reverse transcription-digital droplet PCR (RT-ddPCR) using the standard curve approach. The samples were analyzed by two assays: PVY-uni (45) and the calibrator gene cytochrome oxidase (*COX*) (46). A mix of PVY-infected potato samples was used for the standard curve. The absolute number of copies for each dilution point of the standard curve was determined by the QX100 Droplet Digital PCR system (Bio-Rad) according to a protocol previously described (47), using the same reaction mixture composition and conditions as in the respective qPCRs. The samples were analyzed by RT-qPCR using the AgPath-ID one-step RT-PCR kit (Applied Biosystems) on an ABI 7900 HT Fast instrument (Applied Biosystems) according to a previously described procedure (48), with a manual fluorescence threshold of 0.1 for both assays. Two dilutions (10× and 100×) of isolated RNA, using two technical replicates, were analyzed by both assays to control the dynamics of the replication for each sample. To normalize the different inputs of RNA in reaction mixtures, the determined copy number for PVY-uni was normalized by the copy number of the calibrator gene cytochrome oxidase (*COX*), normalizing the number of PVY copies per 1 copy of *COX*. Results are shown in Data Set S2 in the supplemental material.

Pairwise *t* tests were conducted to test for the differences between the means of the viral titers for the three genotypes (P-D, P-Dn, D-Dn) 21 dpi in the first passage. Pairwise *t* tests were also conducted for the pairs of consecutive passages (1st and 2nd, 2nd and 3rd, 3rd and 4th, 4th and 5th, and 5th and 6th) to test for differences between the mean viral titers determined at 21 dpi. IBM SPSS Statistics version 23 (IBM) was used for these tests.

The number of viral copies in the 250 μl of the inoculum (amount of the inoculum used to infect each plant) was estimated for different lineages in different passages. First, we calculated the average number of *COX* copies in the reaction mixture for all of the measured samples. Since all of the RNA isolations were performed from the leaf discs of the same size (∼45 mg), we then calculated the average number of *COX* copies in 250 mg of leaf tissue. Finally, the number of PVY copies in 250 μl of the inoculum was calculated by multiplying the normalized PVY copy number (an average of two measured plants for each passage/lineage) by the average number of *COX* copies in 250 mg of plant tissue and the dilution coefficient used for the preparation of the inoculum.

### Virus population sequencing.

Small RNA (sRNA) deep sequencing was employed to determine the population structure of selected samples (Fig. 1). Our previous research showed that virus-derived sRNAs accurately describe the diversity of PVY in potato (31).

Plant material sampled 21 dpi or/and inocula were sequenced for the source inoculum and at the 1st, 4th, 5th, and 6th passages in the evolution experiment (Fig. 1). For the 1st and 6th passages, RNA was isolated from the frozen half of the plant material (the other half was used for inoculum preparation) using TRIzol reagent (Invitrogen), according to the manufacturer's instructions for difficult tissue samples, using 600 μl of TRIzol per 100 mg of plant tissue and the FastPrep-24 instrument (MP Biomedicals, USA) for homogenization. To test if small RNA sequencing can be performed on RNA isolated directly from inocula, we isolated RNA simultaneously from the frozen plant material used for the source inoculum and from the source inoculum itself. RNA was isolated from source inoculum using TRIzol LS (Invitrogen) according to the manufacturer protocol for liquid samples. Those two samples were then sequenced, and the results showed that high-quality small RNA libraries could be obtained also from inoculum itself. The determined virus population structures in the two samples were highly similar. Consequently, we decided to sequence inocula directly for the plants of the 4th and 5th passages. Details about each sequenced sample are given in Data Set S1 in the supplemental material.

Due to the narrow genetic bottleneck observed between the 5th and 6th passages of the experiment and consequently a low infection rate, we were not able to obtain PVY-positive samples for NGS for the 6th passage of lineage P1 and the 5th and 6th passages of lineage D1. The plant sample used for sequencing of the 6th passage of lineage D2 was sampled at 44 dpi (Fig. 1).

Total RNA isolated from either plant material or inocula (explained above) was sent for sequencing to SeqMatic LLC (Fremont, CA, USA), where Illumina sRNA libraries were prepared using the TailorMix miRNA Sample Preparation kit (SeqMatic LLC, Fremont, CA, USA). Ten to 13 samples were pooled in one lane of an Illumina HiSeq2000 instrument and sequenced in 1 × 50-bp mode. In each lane used, one of the samples was replicated to allow a later manual determination of the frequency cutoff to distinguish between real SNPs and sequencing/PCR/reverse transcription errors.

### Processing of NGS data.

Sequencing reads were imported in CLC Genomics Workbench 8 (Qiagen). First, quality control and adaptor trimming were performed, and then reads 21 to 24 nucleotides long were selected (representing small RNAs). Those were exported and mapped to the reference genome sequence of PVY (representing the consensus genome sequence of the PVY population from the source inoculum) using the BWA 0.7.8 (49) bwa aln algorithm. The mappings were normalized using samtools 1.1 (50), so that the average depth of coverage amounted to ∼7,500× in all but 3 samples, in which the sequencing depth was lower (Data Set S1). Subsampling some files with higher coverage to comparably lower coverage (5,000×, 2,500×) showed that lower coverage in those samples does not affect the sensitivity of further analyses. Mapping files (BAM) were imported back to CLC Genomics Workbench, where SNP calling was performed using the “low frequency variant detection” tool (significance = 1%; minimum frequency = 0.1%; minimum central quality = 20; minimum neighborhood quality of 5 nucleotides [nt] = 15; forward/reverse ratio > 0.001%). SNP tables were further filtered according to the rules established by a comparison of SNP tables of technical replicates. All of the SNPs present at a frequency of >0.8% were present in both technical replicates in all of the replicate pairs. Using this value as a cutoff, all of the SNPs that were present in a population at least at 0.8% in one of the time points (inspecting each of the 10 lineages independently) were considered true SNPs (even for the time points at which their frequency was lower). Second, we created a rule to determine true SNPs in the source inoculum: in addition to all of the SNPs present at a frequency of >0.8%, SNPs with lower frequencies were also considered true SNPs if they reached a frequency of >0.8% in later time points in at least two independent lineages. All of the SNPs and their frequencies in all of the samples are shown in Data Set S3 in the supplemental material.

### Nucleotide diversity indices (π).

SNP data were used as an input for SNPGenie software (51), which was used to calculate the diversity indices for each population. The consensus sequence of the source inoculum sample was used as a reference. Average whole-genome nucleotide diversities (for all SNPs, synonymous and nonsynonymous) were calculated separately for all SNPs, SNPs already present in inoculum (standing variation), and newly appeared SNPs (new variation). The results were plotted as line plots (Fig. 3) using R 3.2.3 (52). Pairwise *t* tests were conducted using IBM SPSS Statistics version 23 (IBM) to test the significance (*P* < 0.05) of the difference between the means of the *π* values between the 1st and the 4th passages of the evolution experiment. For new variation, all of the lineages were included in the same analysis, whereas for standing variation, lineage DnxD1 was excluded from the analysis (alternating hosts; *π* remained stable for this lineage) and the rest of the lineages were analyzed in two groups (D1, D2, D3, Dn1, Dn2, Dn3, and P2 constituting the first group, with higher *π* values, and P1 and P3 the second, with lower *π* values).

### Wright-Fisher approximate Bayesian calculations.

WF-ABC (19) was used to determine effective population sizes (*N_e_*) of populations and selection coefficients (*s*) of the SNPs. This program uses time-sampled data (frequencies of alleles at consecutive time points) to infer *N_e_* and *s*. In the first step, *N_e_* is calculated using information from all of the observed SNPs in the genome. In the second step, the estimated *N_e_* is used to infer *s* values for each of the observed SNPs separately by using ABC-based simulations of SNP temporal trajectories according to the Wright-Fisher model and finally comparing those simulations to the observed SNP trajectory to infer statistical significance (based on the 95% highest posterior density [HPD] intervals). Accordingly, the program consists of two scripts. The first, wfabc_1, was used to calculate *N_e_* for each lineage. This information was then used as an input for the second script, wfabc_2, which was used to estimate *s* values associated with observed SNPs.

To test if estimates of *N_e_* from SNP frequency data somehow reflect the bottlenecks observed between the 5th and 6th passages by RT-qPCR, we performed wfabc_1 analysis first for all the sequenced time points and then also separately for pairs of time points (inoculum and 1st passage; 1st passage and 4th passage; 4th passage and 5th passage; 5th passage and 6th passage). wfabc_1 was run with the default 10,000 bootstrap replicates, and an average of *N_e_* for all the replicates was calculated.

For the second part of the analysis (selection analysis with wfabc_2), all the time points with observed severe bottlenecks were excluded; thus, the first three passages were used for lineages P2, D1, D2, D3, Dn1, Dn2, and Dn3, and the first four passages for P1 and all 5 passages were used for lineages P3 and DnxD1.

wfabc_2 was run in haploid mode (-ploidy 1) with 100,000 simulations and 1% acceptance rate, setting uniform prior for *s* between −0.5 and 0.5. Using R 3.2.3, the posterior mean, median, and 95% HPD intervals were calculated for each locus. We considered a SNP to be nonneutral if the 95% HPD interval for *s* excluded zero.

The results of the selection analysis were plotted using R 3.2.3. The distributions of posterior means for *s* values for each lineage were plotted as histograms (Fig. 4, right panels). The posterior mean value of estimated *s* for each SNP was plotted on PVY genome coordinates separately for each lineage (Fig. 4, left panels). SNPs with rejected neutrality (95% highest posterior density interval does not contain 0), SNPs assigned to different clusters (see “Analysis of SNP trajectories and clustering” below), synonymous/nonsynonymous mutations, and new/standing mutations are shown in this plot using different symbols and colors.

### Analysis of SNP trajectories and clustering.

The SNPs' trajectories were assigned into groups using the R 3.2.3 (heatmap.2 library) hierarchical clustering approach (method = average). Only the SNPs reaching 5% in at least one time point were used for this analysis, since the SNPs with lower frequencies were not informative for cluster analysis. First, SNP trajectories were clustered for each lineage separately. SNP was treated as a vector with *n* dimensions, dimensions representing the frequencies of the SNP in the sequenced time points. The heatmap.2 library was used to cluster SNPs' trajectories and visualize them as a heat map (Fig. S1).

Second, results of selection analysis were used to cluster both SNPs and lineages. Only the SNPs occurring in all lineages were used for this analysis. Each lineage was treated as a vector with *n* dimensions, dimensions representing the selection coefficients of the observed SNPs. Conversely, each SNP was treated as a vector with *n* dimensions, dimensions representing selection coefficients of the SNP in each lineage. The R 3.2.3 heatmap.2 library was used to cluster both lineages and SNPs and to visualize the *s* values in the form of a heat map. Library pvclust (53) was used to assess the statistical support of clusters using 1,000 bootstrap replicates.

To confirm the results of the hierarchical clustering approach for the observed lineages' clustering, a principal-component analysis (PCA) was performed, reducing the dimensionality of data to major components of variance. R 3.2.3 was used to perform the analysis using the “prcomp” function. The first three principal components of variance were plotted, and the grouping of lineages was visually inspected.

### Accession number(s).

Raw NGS read files (fastq) from this experiment have been deposited under NCBI Sequence Read Archive (SRA) accession number SRP083943 or can be found under BioProject number PRJNA340976. The consensus viral genome sequence from the source inoculum was deposited in NCBI GenBank under accession number KX856986.

## Supplementary Material

Supplemental material
