# Supplementary material for: Time-Sampled Population Sequencing Reveals the Interplay of Selection and Genetic Drift in Experimental Evolution of Potato Virus Y
Source: J Virol. 2017 Jul 27;91(16):e00690-17. doi: 10.1128/JVI.00690-17 (PMC5533922; doi:10.1128/JVI.00690-17)
Supplement: Supplemental material [file supp_91_16_e00690-17__index.html]

Supplemental material 

# Time-Sampled Population Sequencing Reveals the Interplay of Selection and Genetic Drift in Experimental Evolution of *Potato Virus Y*

## Supplemental material

- Supplemental file 1 -

  Data Set S1 (Samples included in population sequencing and their metadata.)

  XLS, 34K
- Supplemental file 2 -

  Data Set S2 (Detailed results of the virus titer measurements using RT-qPCR.)

  XLS, 75K
- Supplemental file 3 -

  Data Set S3 (Variants detected in the experiment and their frequencies in the analyzed samples.)

  XLS, 143K
- Supplemental file 4 -

  Fig. S1 (Clustering of SNP trajectories.)

  PDF, 470K
