# Supplementary material for: Time-Sampled Population Sequencing Reveals the Interplay of Selection and Genetic Drift in Experimental Evolution of Potato Virus Y
Source: J Virol. 2017 Jul 27;91(16):e00690-17. doi: 10.1128/JVI.00690-17 (PMC5533922; doi:10.1128/JVI.00690-17)
Supplement: Supplemental material [file JVI.00690-17_zjv999182822s4.pdf]

Supplementary figure 1: Clustering of SNPs trajectories. The SNP trajectories were assigned into groups using R 3.2.3 (heatmap.2 library) hierarchical clustering approach (method=average). Only the variants reaching 5% in at least one time point were used for this analysis, since the variants with lower frequencies were not informative for cluster analysis. SNPs trajectories were clustered for each lineage separately (a-j). SNP was treated as a vector with *n* dimensions, dimensions representing frequency of the variant in the sequenced time points. Heatmap.2 library was used to cluster SNPs trajectories and visualize them as a heatmap. SNPs labels are colored according to the groups defined in the analysis shown in the main text on Fig. 5. Reconstructed haplotypes are boxed. Black squares next to the SNPs labels designate nonsynonymous mutations.

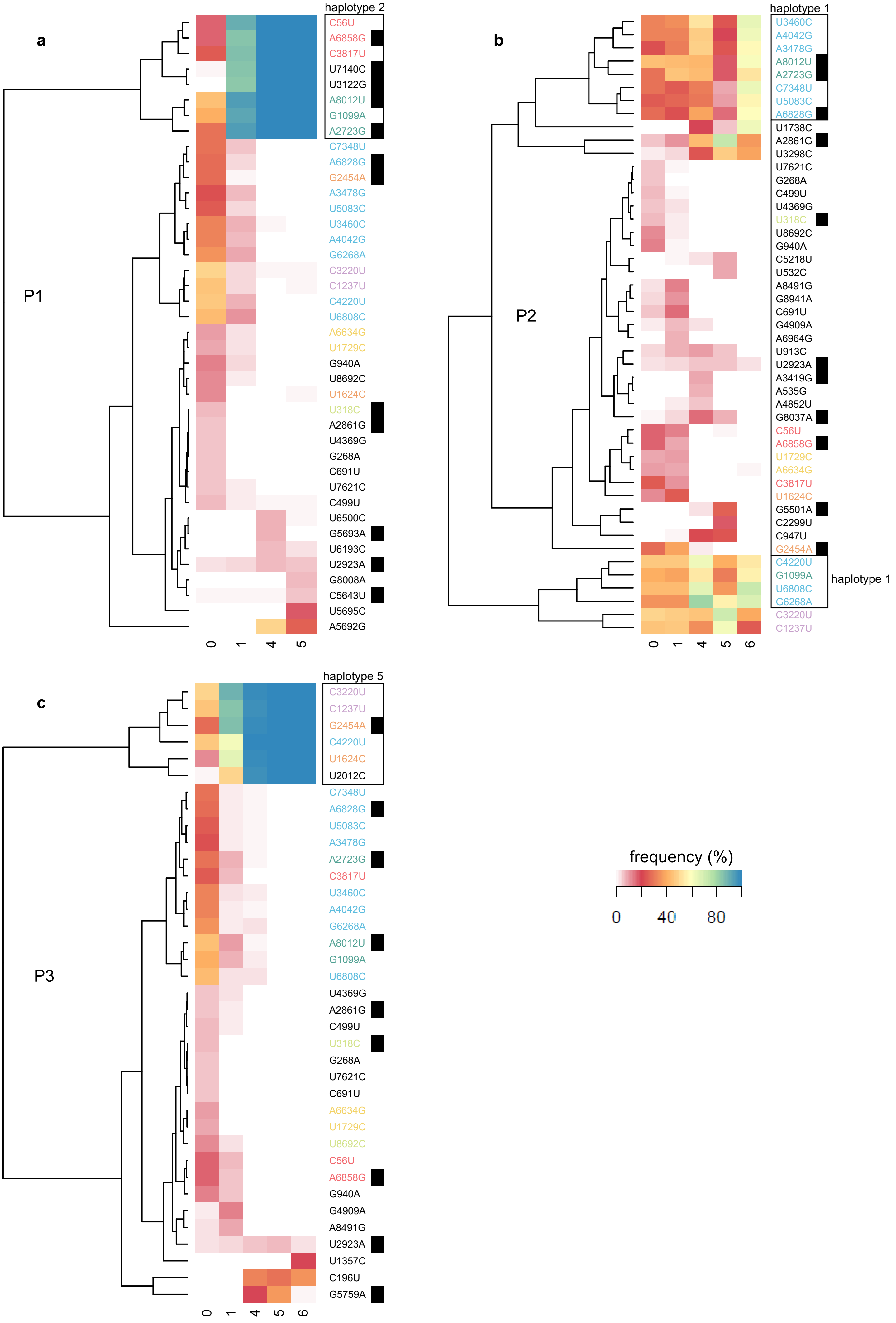

a
P1
haplotype 2
C56U
A6858G
C3817U
U7140C
U3122G
A8012U
G1099A
A2723G
C7348U
A6828G
G2454A
A3478G
U5083C
U3460C
A4042G
G6268A
C3220U
C1237U
C4220U
U6808C
A6634G
U1729C
G940A
U8692C
U1624C
U318C
A2861G
U4369G
G268A
C691U
U7621C
C499U
U6500C
G5693A
U6193C
U2923A
G8008A
C5643U
U5695C
A5692G
0
1
4
5
b
P2
haplotype 1
U3460C
A4042G
A3478G
A8012U
A2723G
C7348U
U5083C
A6828G
U1738C
A2861G
U3298C
U7621C
G268A
C499U
U4369G
U318C
U8692C
G940A
C5218U
U532C
A8491G
G8941A
C691U
G4909A
A6964G
U913C
U2923A
A3419G
A535G
A4852U
G8037A
C56U
A6858G
U1729C
A6634G
C3817U
U1624C
G5501A
C2299U
C947U
G2454A
C4220U
G1099A
U6808C
G6268A
C3220U
C1237U
haplotype 1
0
1
4
5
6
c
P3
haplotype 5
C3220U
C1237U
G2454A
C4220U
U1624C
U2012C
C7348U
A6828G
U5083C
A3478G
A2723G
C3817U
U3460C
A4042G
G6268A
A8012U
G1099A
U6808C
U4369G
A2861G
C499U
U318C
G268A
U7621C
C691U
A6634G
U1729C
U8692C
C56U
A6858G
G940A
G4909A
A8491G
U2923A
U1357C
C196U
G5759A
0
1
4
5
6
frequency (%)
0
40
80

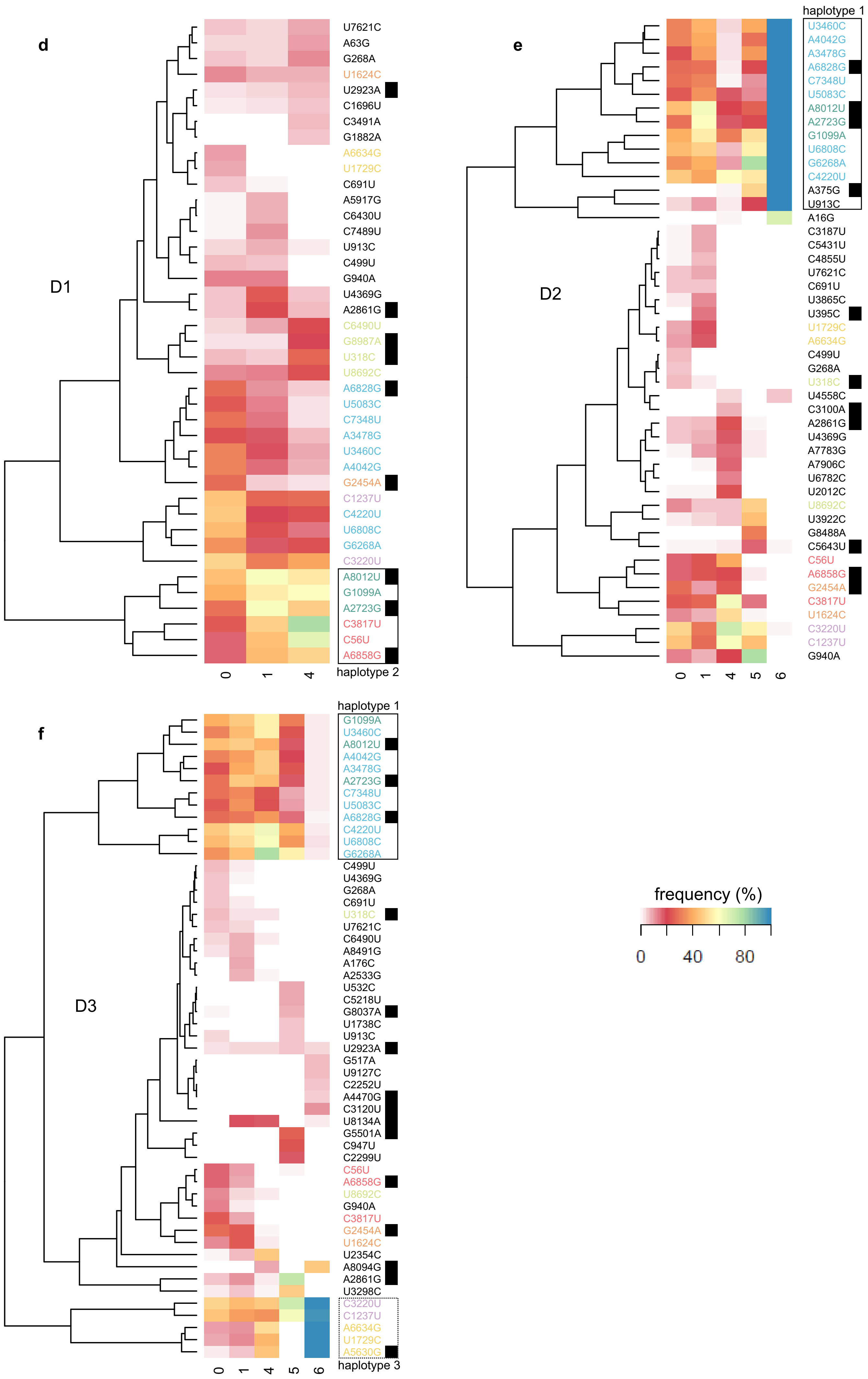
d
D1
U7621C
A63G
G268A
U1624C
U2923A
C1696U
C3491A
G1882A
A6634G
U1729C
C691U
A5917G
C6430U
C7489U
U913C
C499U
G940A
U4369G
A2861G
C6490U
G8987A
U318C
U8692C
A6828G
U5083C
C7348U
A3478G
U3460C
A4042G
G2454A
C1237U
C4220U
U6808C
G6268A
C3220U
A8012U
G1099A
A2723G
C3817U
C56U
A6858G
0
1
4
haplotype 2
e
D2
haplotype 1
U3460C
A4042G
A3478G
A6828G
C7348U
U5083C
A8012U
A2723G
G1099A
U6808C
G6268A
C4220U
A375G
U913C
A16G
C3187U
C5431U
C4855U
U7621C
C691U
U3865C
U395C
U1729C
A6634G
C499U
G268A
U318C
U4558C
C3100A
A2861G
U4369G
A7783G
A7906C
U6782C
U2012C
U8692C
U3922C
G8488A
C5643U
C56U
A6858G
G2454A
C3817U
U1624C
C3220U
C1237U
G940A
0
1
4
5
6
f
D3
haplotype 1
G1099A
U3460C
A8012U
A4042G
A3478G
A2723G
C7348U
U5083C
A6828G
C4220U
U6808C
G6268A
C499U
U4369G
G268A
C691U
U318C
U7621C
C6490U
A8491G
A176C
A2533G
U532C
C5218U
G8037A
U1738C
U913C
U2923A
G517A
U9127C
C2252U
A4470G
C3120U
U8134A
G5501A
C947U
C2299U
C56U
A6858G
U8692C
G940A
C3817U
G2454A
U1624C
U2354C
A8094G
A2861G
U3298C
C3220U
C1237U
A6634G
U1729C
A5630G
0
1
4
5
6
haplotype 3
frequency (%)
0
40
80

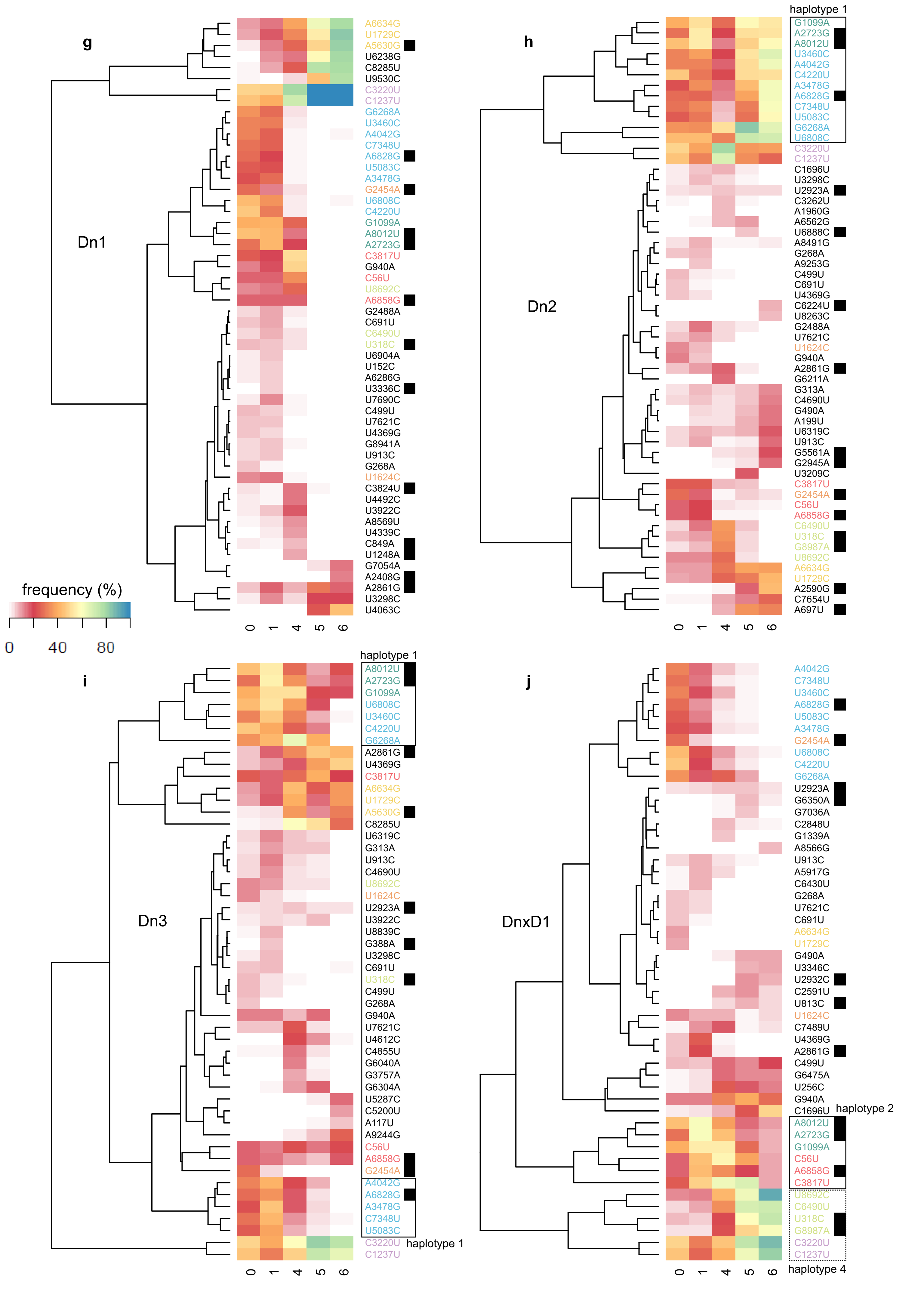
g
Dn1
A6634G
U1729C
A5630G
U6238G
C8285U
U9530C
C3220U
C1237U
G6268A
U3460C
A4042G
C7348U
A6828G
U5083C
A3478G
G2454A
U6808C
C4220U
G1099A
A8012U
A2723G
C3817U
G940A
C56U
U8692C
A6858G
G2488A
C691U
C6490U
U318C
U6904A
U152C
A6286G
U3336C
U7690C
C499U
U7621C
U4369G
G8941A
U913C
G268A
U1624C
C3824U
U4492C
U3922C
A8569U
U4339C
C849A
U1248A
G7054A
A2408G
A2861G
U3298C
U4063C
0
1
4
5
6
frequency (%)
0
40
80
h
Dn2
haplotype 1
G1099A
A2723G
A8012U
U3460C
A4042G
C4220U
A3478G
A6828G
C7348U
U5083C
G6268A
U6808C
C3220U
C1237U
C1696U
U3298C
U2923A
C3262U
A1960G
A6562G
U6888C
A8491G
G268A
A9253G
C499U
C691U
U4369G
C6224U
U8263C
G2488A
U7621C
U1624C
G940A
A2861G
G6211A
G313A
C4690U
G490A
A199U
U6319C
U913C
G5561A
G2945A
U3209C
C3817U
G2454A
C56U
A6858G
C6490U
U318C
G8987A
U8692C
A6634G
U1729C
A2590G
C7654U
A697U
0
1
4
5
6
i
Dn3
haplotype 1
A8012U
A2723G
G1099A
U6808C
U3460C
C4220U
G6268A
A2861G
U4369G
C3817U
A6634G
U1729C
A5630G
C8285U
U6319C
G313A
U913C
C4690U
U8692C
U1624C
U2923A
U3922C
U8839C
G388A
U3298C
C691U
U318C
C499U
G268A
G940A
U7621C
U4612C
C4855U
G6040A
G3757A
G6304A
U5287C
C5200U
A117U
A9244G
C56U
A6858G
G2454A
A4042G
A6828G
A3478G
C7348U
U5083C
C3220U
haplotype 1
C1237U
0
1
4
5
6
j
DnxD1
A4042G
C7348U
U3460C
A6828G
U5083C
A3478G
G2454A
U6808C
C4220U
G6268A
U2923A
G6350A
G7036A
C2848U
G1339A
A8566G
U913C
A5917G
C6430U
G268A
U7621C
C691U
A6634G
U1729C
G490A
U3346C
U2932C
C2591U
U813C
U1624C
C7489U
U4369G
A2861G
C499U
G6475A
U256C
G940A
C1696U
haplotype 2
A8012U
A2723G
G1099A
C56U
A6858G
C3817U
U8692C
C6490U
U318C
G8987A
C3220U
C1237U
haplotype 4
0
1
4
5
6
